# Supplementary material for: Effect of Fibroblast Growth Factor 10 and an Interacting Non-Coding RNA on Secondary Hair Follicle Dermal Papilla Cells in Cashmere Goats’ Follicle Development Assessed by Whole-Transcriptome Sequencing Technology
Source: Animals (Basel). 2023 Jul 7;13(13):2234. doi: 10.3390/ani13132234 (PMC10340072; doi:10.3390/ani13132234)
Supplement: Supplementary file 1 [file animals-13-02234-s001.zip › Supplementary Figure S1.pdf]

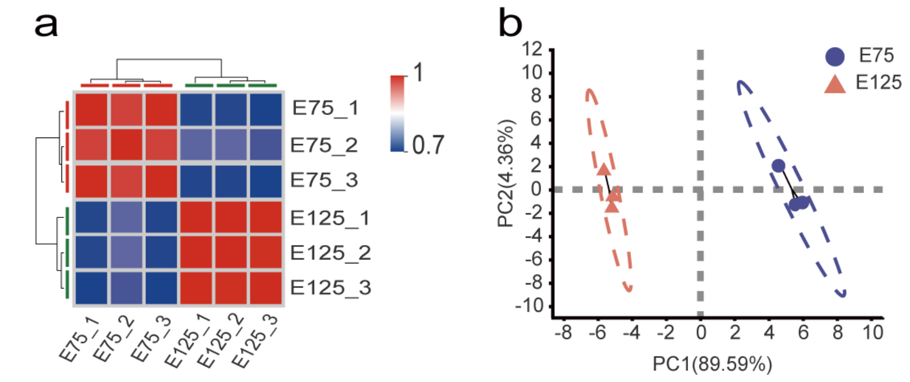

Figure S1 (a) Correlation analysis. The clustering of each group is shown on the left and top. The different colour squares represent the high correlation between the two samples, where larger values indicate a higher correlation between the two samples. (b) Principal component analysis (PCA). The distance of each sample point represents the distance between samples; the closer the distance the higher the similarity between samples, indicating better biological replication between samples.
