# Supplementary material for: Effect of Fibroblast Growth Factor 10 and an Interacting Non-Coding RNA on Secondary Hair Follicle Dermal Papilla Cells in Cashmere Goats’ Follicle Development Assessed by Whole-Transcriptome Sequencing Technology
Source: Animals (Basel). 2023 Jul 7;13(13):2234. doi: 10.3390/ani13132234 (PMC10340072; doi:10.3390/ani13132234)
Supplement: Supplementary file 1 [file animals-13-02234-s001.zip › Supplementary Figure S2.pdf]

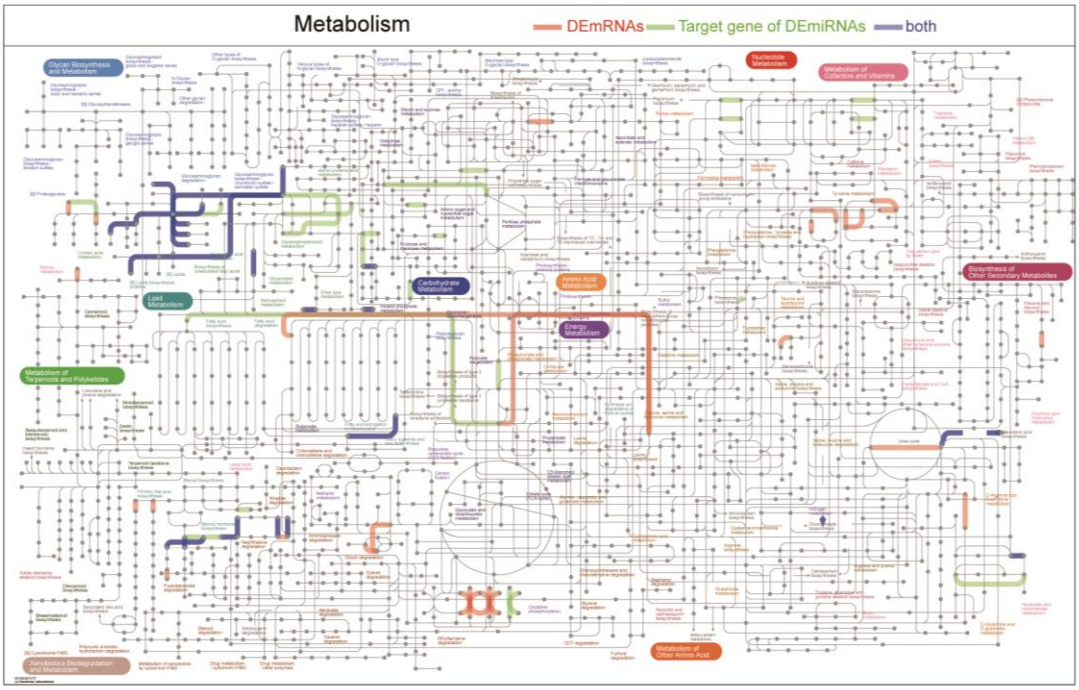

Figure S2 Analysis of the ipath metabolic pathway. Metabolism represents the complete metabolic system of the biological system. Red, light green, and blue represent pathways annotated by the gene sets of DEmRNAs, the target gene sets of DEmiRNAs, and pathways annotated by the genes in both gene sets, respectively.
