# Supplementary material for: Effect of Fibroblast Growth Factor 10 and an Interacting Non-Coding RNA on Secondary Hair Follicle Dermal Papilla Cells in Cashmere Goats’ Follicle Development Assessed by Whole-Transcriptome Sequencing Technology
Source: Animals (Basel). 2023 Jul 7;13(13):2234. doi: 10.3390/ani13132234 (PMC10340072; doi:10.3390/ani13132234)
Supplement: Supplementary file 1 [file animals-13-02234-s001.zip › Supplementary Figure S3 .pdf]

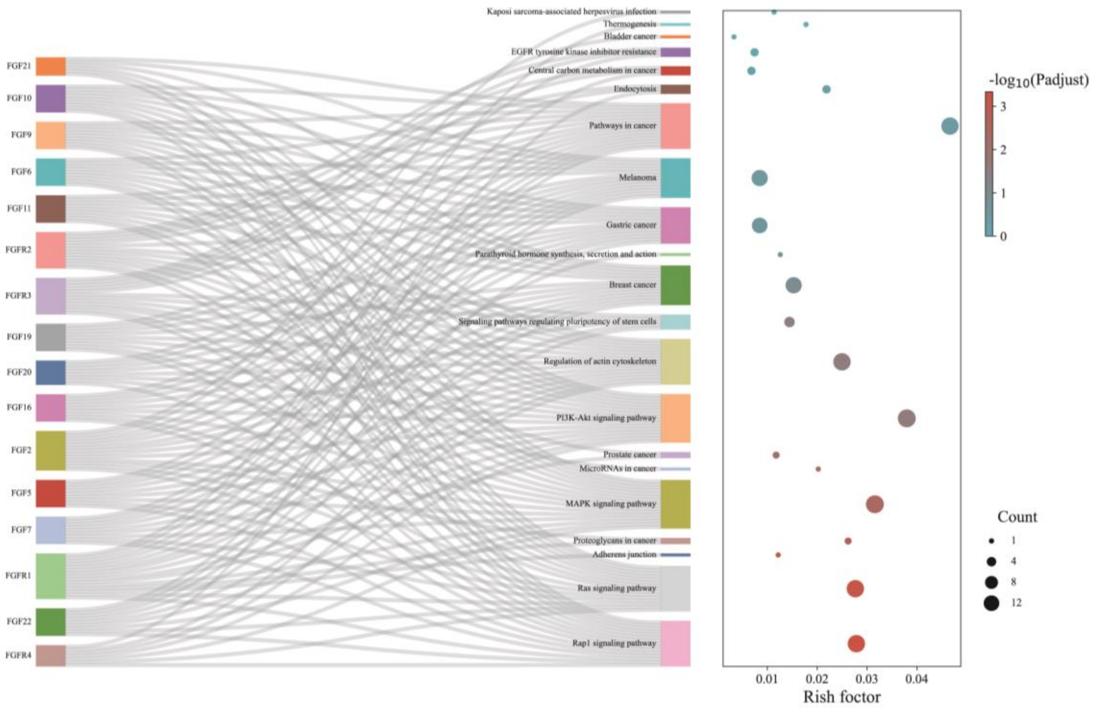

Figure S3 Sankey bubble map of the FGF family. To further understand the role of the FGF family in hair follicle development, we generated a collection of transcripts of the differential FGF family and its receptors, and enrichment analysis showed that the family is highly enriched in cancer-related pathways and plays a role in the regulation of stem cell pluripotency, The enrichment of MAPK, PI3K-AKT, RAS, RAP1 and other pathways related to hair follicle development and regeneration suggests that the FGF family and its receptors are involved in the regulation of hair follicle processes.
