# Supplementary material for: Effect of Fibroblast Growth Factor 10 and an Interacting Non-Coding RNA on Secondary Hair Follicle Dermal Papilla Cells in Cashmere Goats’ Follicle Development Assessed by Whole-Transcriptome Sequencing Technology
Source: Animals (Basel). 2023 Jul 7;13(13):2234. doi: 10.3390/ani13132234 (PMC10340072; doi:10.3390/ani13132234)
Supplement: Supplementary file 1 [file animals-13-02234-s001.zip › Supplementary Figure S4.pdf]

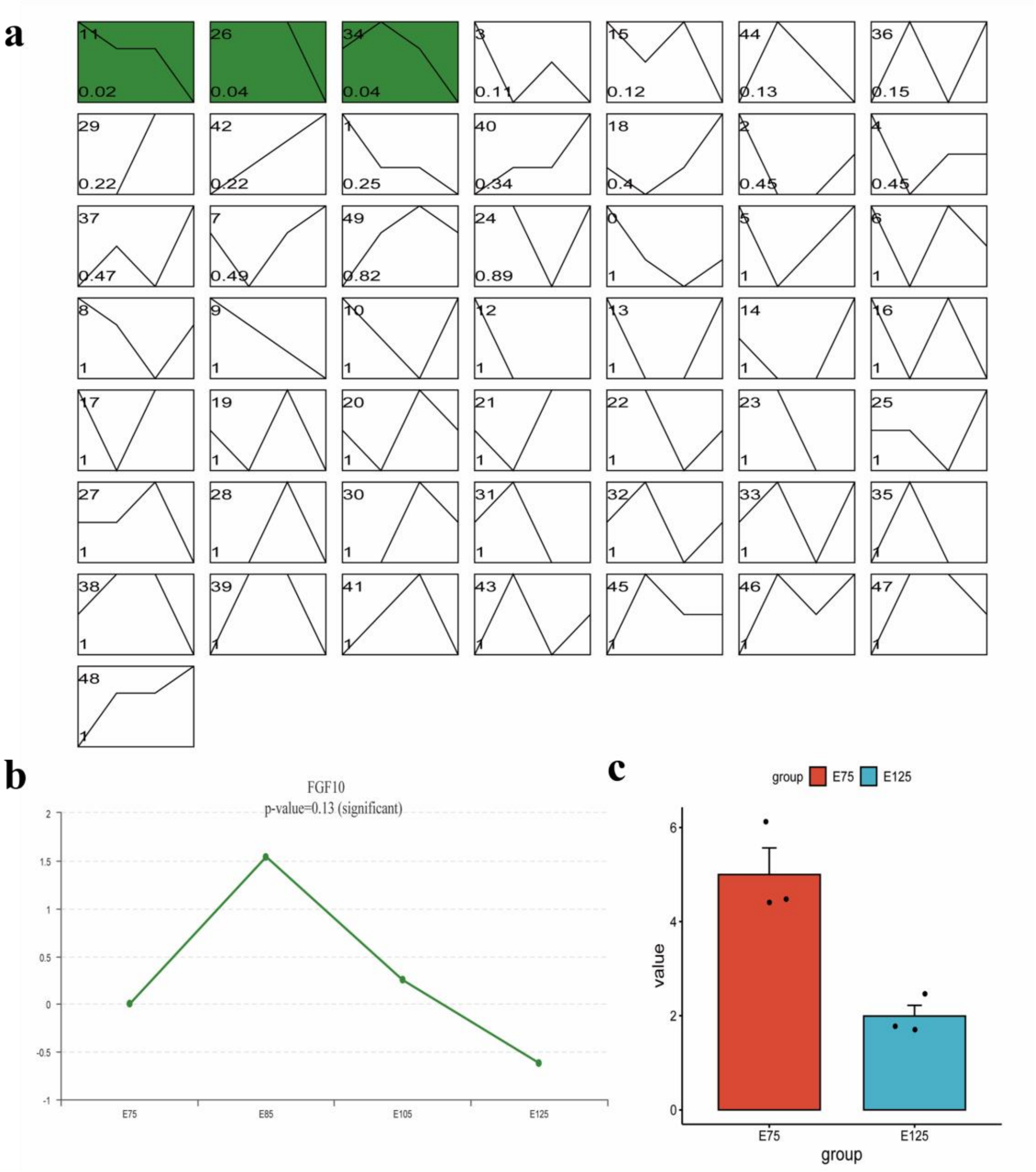

Figure S4 FGF10 expression analysis. (**a**) Temporal analysis of the differential FGF family and its receptor transcript collection showed significant enrichment in three expression patterns (P<0.05); (**b**) dynamic trends in FGF10 expression; (**c**) differential expression analysis showed that FGF10 was more highly expressed at E75 than at E125, and the differential expression was significant.
