# Supplementary material for: Effect of Fibroblast Growth Factor 10 and an Interacting Non-Coding RNA on Secondary Hair Follicle Dermal Papilla Cells in Cashmere Goats’ Follicle Development Assessed by Whole-Transcriptome Sequencing Technology
Source: Animals (Basel). 2023 Jul 7;13(13):2234. doi: 10.3390/ani13132234 (PMC10340072; doi:10.3390/ani13132234)
Supplement: Supplementary file 1 [file animals-13-02234-s001.zip › Supplementary Table S1.pdf]

Table S1 Primers for vectors construction

Name	Primer (5'-3')
FGF10	F:cgcGGATCCATGTGGAAATGGATTCTGAC
	R:ccgGAATTCCTATGAGTGTACCACCATTG
siRNA-*FGF10*-220	F:GUUGCUGUGAAAGCUAUUATT
	R:UAAUAGCUUUCACAGCAACTT
siRNA-*FGF10*-273	F:CCAAGUACUUUCUCAAGAUTT
	R:AUCUUGAGAAAGUACUUGGTT
siRNA-*FGF10*-374	F:GAGCUACAAUCACCUCCAATT
	R:UUGGAGGUGAUUGUAGCUCTT
FGF10-siRNA-NC	F:UUCUCCGAACGUGUCACGUTT
	R:ACGUGACACGUUCGGAGAATT
miR-184 mimics	F:UGGACGGAGAACUGAUAAGGGU
	R:CCUUAUCAGUUCUCCGUCCAUU
miR-184 mimics NC	F:UUCUCCGAACGUGUCACGUTT
	R:ACGUGACACGUUCGGAGAATT
miR-184 inhibitors	F:ACCCUUAUCAGUUCUCCGUCCA
miR-184 inhibitors NC	F:CAGUACUUUUGUGUAGUACAA
