# Supplementary material for: Effect of Fibroblast Growth Factor 10 and an Interacting Non-Coding RNA on Secondary Hair Follicle Dermal Papilla Cells in Cashmere Goats’ Follicle Development Assessed by Whole-Transcriptome Sequencing Technology
Source: Animals (Basel). 2023 Jul 7;13(13):2234. doi: 10.3390/ani13132234 (PMC10340072; doi:10.3390/ani13132234)
Supplement: Supplementary file 1 [file animals-13-02234-s001.zip › Supplementary Table S2.pdf]

Table S2 Primers for double luciferase reporter gene vectors

| Name | Primer (5'-3') |
|---|---|
| circRNA-0001141-WT | F:TCGAAGGCCAACACUGGAAUCCGUCCG<br>R:CTAGCGGACGGATTCCAGTGTTGGCCT |
| circRNA-0001141-MUT | F:TCGAAGGCCAACACUGGAACATAUAAG<br>R:CTAGCTTATATGTTCCAGTGTTGGCCT |
| *FGF10*-WT-1 | F:TCGAGCTCTTCTTGGTGTCTTCCGTCCC<br>R:CTAGGGGACGGAAGACACCAAGAAGAGC |
| *FGF10*-MUT-1 | F: TCGAGGTGAACATGCAGAGTAGGCAGGC<br>R:CTAGGCCTGCCTACTCTGCATGTTCACC |
| *FGF10*-WT-2 | F:TCGATATCCTCAGCAGCTGCTCTCTGTTT<br>R:CTAGAAACAGAGAGCAGCTGCTGAGGATA |
| *FGF10*-MUT-2 | F:TCGATTAGGACAGGTCCTGCAGAGACAAT<br>R:CTAGATTGTCTCTGCAGGACCTGTCCTAA |
