# Supplementary material for: Effect of Fibroblast Growth Factor 10 and an Interacting Non-Coding RNA on Secondary Hair Follicle Dermal Papilla Cells in Cashmere Goats’ Follicle Development Assessed by Whole-Transcriptome Sequencing Technology
Source: Animals (Basel). 2023 Jul 7;13(13):2234. doi: 10.3390/ani13132234 (PMC10340072; doi:10.3390/ani13132234)
Supplement: Supplementary file 1 [file animals-13-02234-s001.zip › Supplementary Table S3.pdf]

Table S3 longRNA-seq sequencing data statistics

Sample	Raw reads	Clean reads	N(%)	Q20(%)	Q30(%)	GC(%)
E75-1	130674098	129541578	0.0235	98.63	95.72	55.13
E75-2	128856140	127880120	0.0236	98.59	95.63	53.99
E75-3	130751966	129819626	0.0238	98.52	95.42	54.19
E125-1	144366422	143366824	0.0235	98.65	95.7	51.71
E125-2	142303864	141260588	0.0237	98.55	95.47	52.67
E125-3	136188780	135220562	0.0237	98.56	95.5	53.39
