# Supplementary material for: Effect of Fibroblast Growth Factor 10 and an Interacting Non-Coding RNA on Secondary Hair Follicle Dermal Papilla Cells in Cashmere Goats’ Follicle Development Assessed by Whole-Transcriptome Sequencing Technology
Source: Animals (Basel). 2023 Jul 7;13(13):2234. doi: 10.3390/ani13132234 (PMC10340072; doi:10.3390/ani13132234)
Supplement: Supplementary file 1 [file animals-13-02234-s001.zip › Supplementary Table S4.pdf]

Table S4 smallRNA-seq sequencing data statistics

Sample	Raw reads	Clean reads	N(%)	Q20(%)	Q30(%)	GC(%)
E75-1	14513270	14177178	0.0232	98.77	95.96	42.71
E75-2	15997736	15455878	0.023	98.84	96.22	43.56
E75-3	15372318	14977603	0.0237	98.46	95.5	42.76
E125-1	14629622	14066324	0.0236	98.58	95.58	44.93
E125-2	14110152	13675726	0.0232	98.76	95.98	44.95
E125-3	17292026	16692307	0.0231	98.77	96.15	45.36
