# Supplementary material for: Effect of Fibroblast Growth Factor 10 and an Interacting Non-Coding RNA on Secondary Hair Follicle Dermal Papilla Cells in Cashmere Goats’ Follicle Development Assessed by Whole-Transcriptome Sequencing Technology
Source: Animals (Basel). 2023 Jul 7;13(13):2234. doi: 10.3390/ani13132234 (PMC10340072; doi:10.3390/ani13132234)
Supplement: Supplementary file 1 [file animals-13-02234-s001.zip › Supplementary Table S6.pdf]

Table S6 Primer sequences for qRT-PCR

ID	Name	Primer (5'-3')	Product size
ENSCHIG00010000007	*U6*	F：GCTTCGGCAGCACATATACTAAAAT<br>R：CGCTTCACGAATTTGCGTGTCAT	50
-	*U6* RT	CGCTTCACGAATTTGCGTGTCAT	-
ENSCHIG00000013316	*β-actin*	F：ATCGTGATGGACTCCGGTGA<br>R：CCACGCTCCGTGAGAATCTT	140
ENSCHIG00000007281	*klf13*	F：GGGCTGCGAGAAAGTTTACG<br>R：GACGCTCTCCTGTGTGAGTT	71
ENSCHIG00000009242	*VEGFA*	F：TGCGGATCAAACCTCACCAA<br>R：CCTCGGCTTGTCACATCTTTC	119
ENSCHIG00000021319	*Col1a1*	F：AGCCCTGGTGAAAATGGAGC<br>R：TTCACCCTTAGCACCCACAG	192
ENSCHIG00000001082	*KRT5*	F：ACGTCTGCTGAGGGAGTACC<br>R：AGCCTGAGGAAACGGTGTTTG	164
chi_circ_0011012	*chi_circ_0011012*	F：ACCATGGCCAACAATTCTGC<br>R：GTCTGGGACCCGTATGGCTG	162
chi_circ_0065827	*chi_circ_0065827*	F：AGGGGATGGGTGTGGATGTAA<br>R：GAGAGTGGGGGAATGAGACAC	199
chi_circ_0000227	*chi_circ_0000227*	F：GTCCCTCCTGCACCAAGAAA<br>R：AGGGCTCAAACACCCAGATT	179
chi_circ_0035645	*chi_circ_0035645*	F：TGTCTCTCAAGGCTGCTGTC<br>R：AGGGCATAATACAGTCACACCA	117
ENSCHIG00010017178	*FGF10*	F：TGTCTCTCAAGGCTGCTGTC<br>R：AGGGCATAATACAGTCACACCA	117

Bulge-loop^{TM} miRNA qRT-PCR primers (each set consists of one reverse primer and one pair of qPCR primers) for miR-134, miR-24-3p, miR-214-3p, miR-200a, let-7b-5p and miR-184 expression assays were synthesized by Reebok.
